# Supplementary material for: The SARS-CoV-2 Nsp3 macrodomain reverses PARP9/DTX3L-dependent ADP-ribosylation induced by interferon signaling
Source: J Biol Chem. 2021 Aug 4;297(3):101041. doi: 10.1016/j.jbc.2021.101041 (PMC8332738; doi:10.1016/j.jbc.2021.101041)
Supplement: Supplemental Figures S1–S5 and Table S1 [file mmc1.pdf]

Supplementary Figure 1

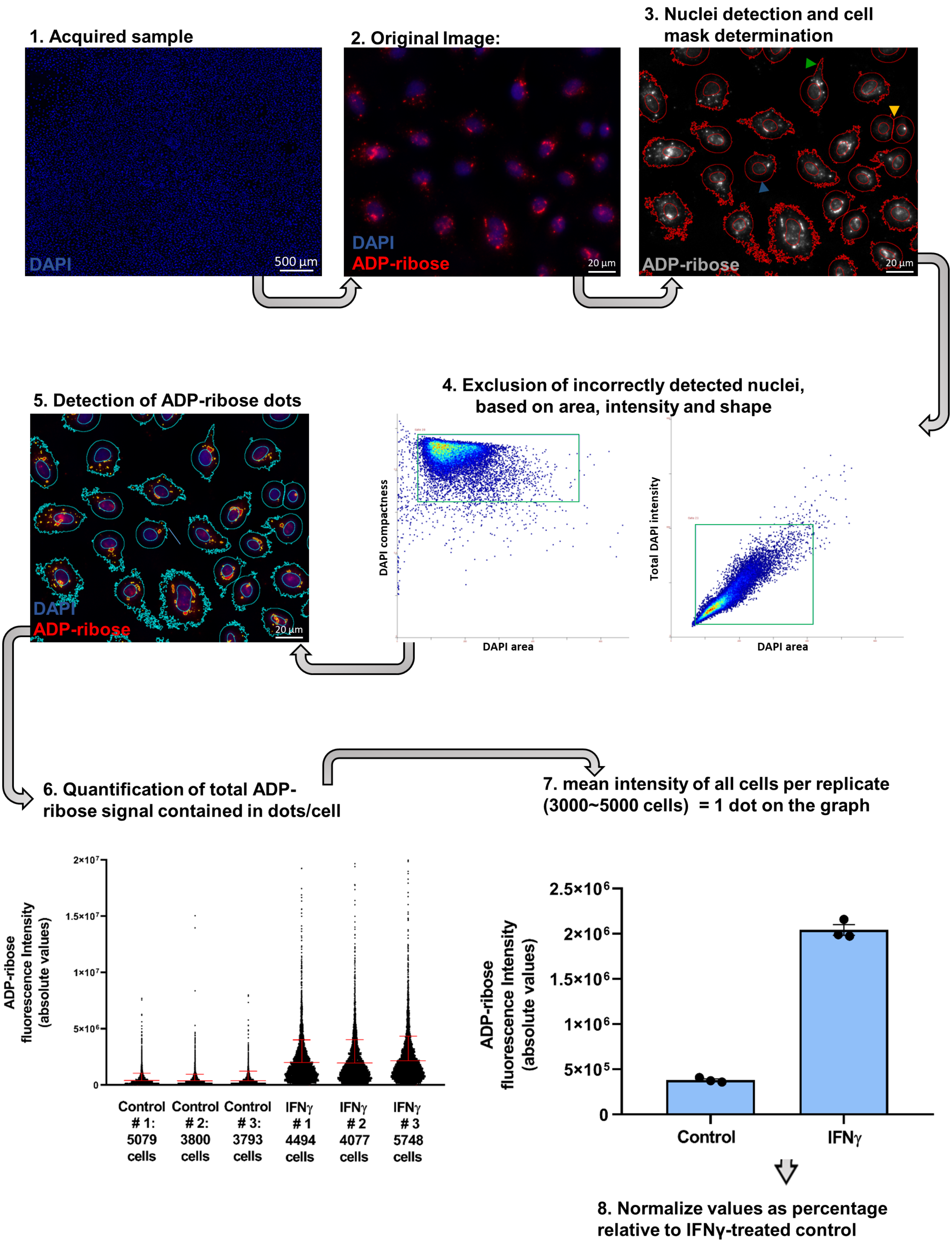

Supplementary Figure 1 – Image Analysis Pipeline

1. Representative image of a complete sample acquire for a single well (DAPI channel only). Individual fields of view were stitched to form the shown image. Scale bar = 500 μm

2. Representative image of a small portion of the sample, showing DAPI (blue) and ADP-ribose signal (red) in cells treated with IFNγ. Scale bar = 20 μm

3. Representative image of the same cells as in 2, showing the ADP-ribose signal (grey) and the ring mask area in which ADP-ribose signal was quantified. Inner red circle is based on nuclei detection in the DAPI channel, outer red limit is either grown outwards from the nucleus by a fixed distance (blue arrow), until neighbouring areas touch each other (yellow arrow) or until ADP-ribose signal reached background values (green arrow). Scale bar = 20 μm

4. Scatterplots of DAPI area x Total DAPI intensity (right) or DAPI area x DAPI compactness (a measure of "roundness" of the nucleus) (left) for all nuclei detected in image 1. Each dot represents one nucleus, with clustering of nuclei represented by colour changes. Only nuclei within the shown gates were taken forward for quantification, as outliers were either incompletely acquired or not properly detected. Scale bar = 20μm

5. Representative image of the same cells as in 2, showing DAPI (blue), ADP-ribose signal (red), the ring mask area from step 3 (light green) and the detection of ADP-ribose dots (marked in yellow).

6. Representative column scatter plots of total ADP-ribose signal intensity contained in ADP-ribose dots per cell for three replicate samples performed in one experiment. Numbers of cells per sample are shown below each replicate. Each black dot represents one cell, red bars are mean + SEM.

7. Representative bar graph of ADP-ribose signal intensities. The mean signal intensity from 3000 to 5000 cells per replicate (red bar in graph 6) is plotted as a point, with the mean between replicates shown as a bar ± SEM.

8. For all figures in the manuscript, absolute signal intensities were normalized to the respective IFNγ-treated control (considered to be 100%), to normalize for variations in staining intensities between replicate experiments.

Supplementary Figure 2

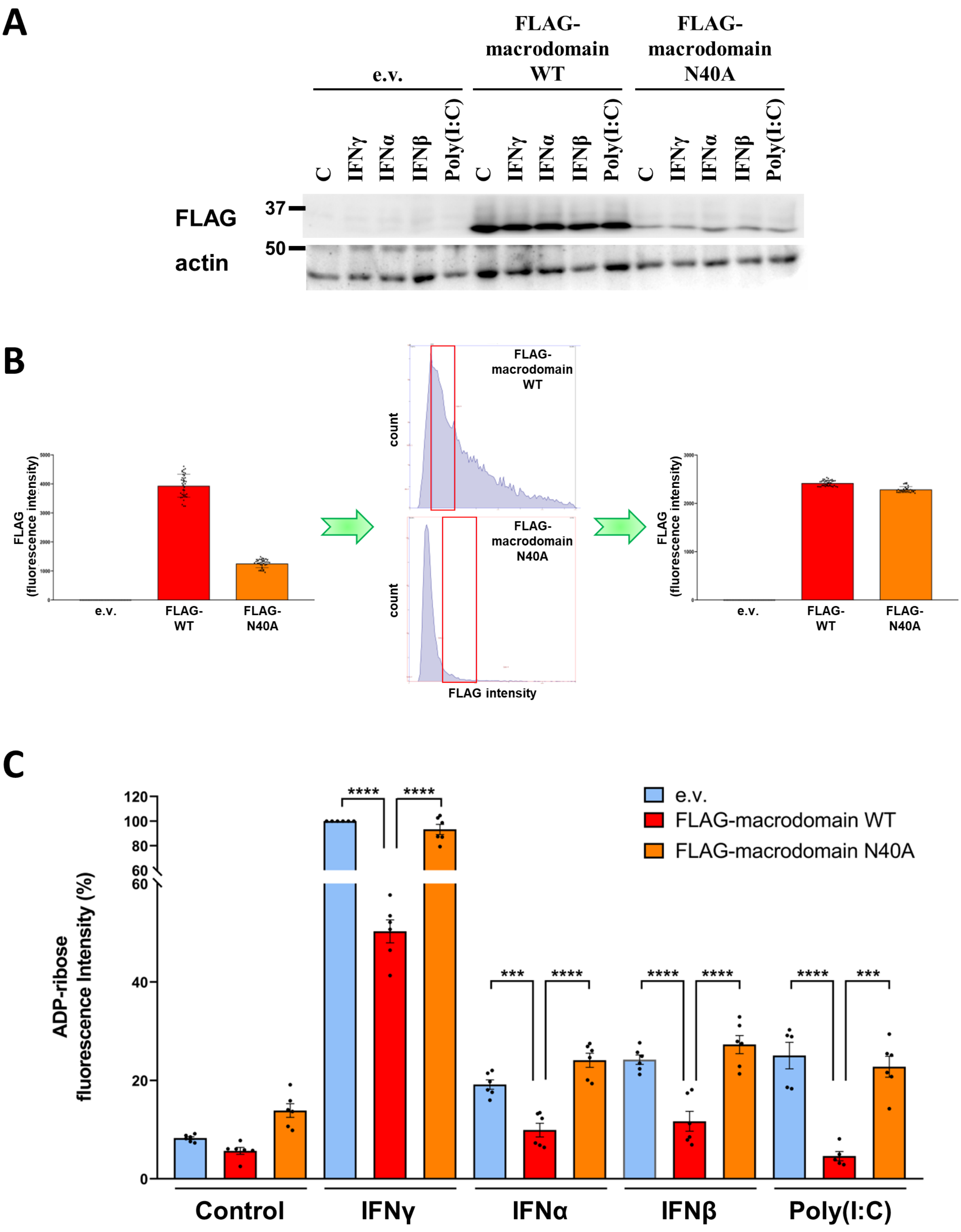

Supplementary Figure 2

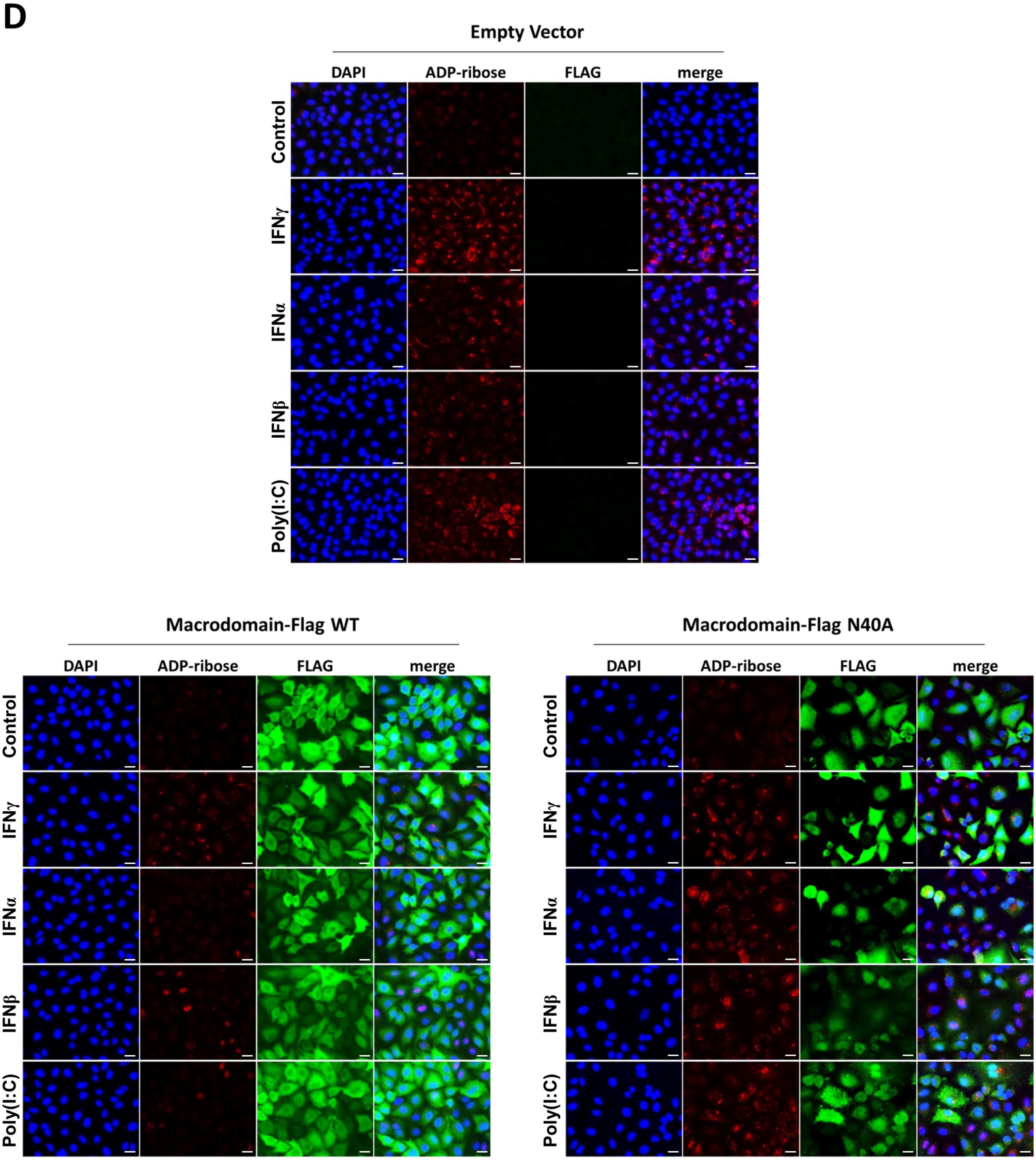

Supplementary Figure 2

E

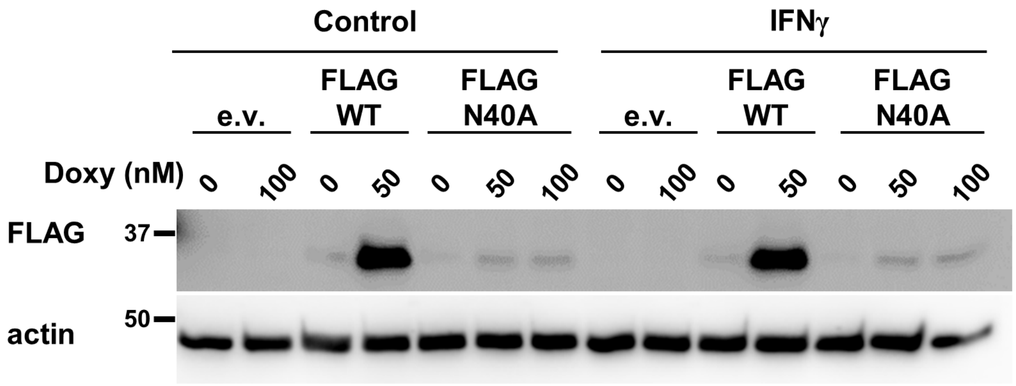

F

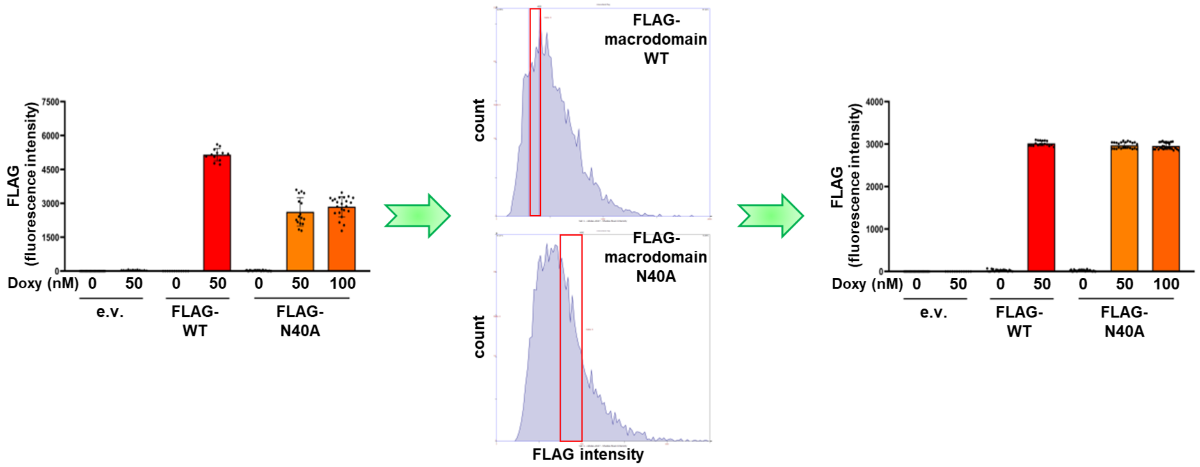

G

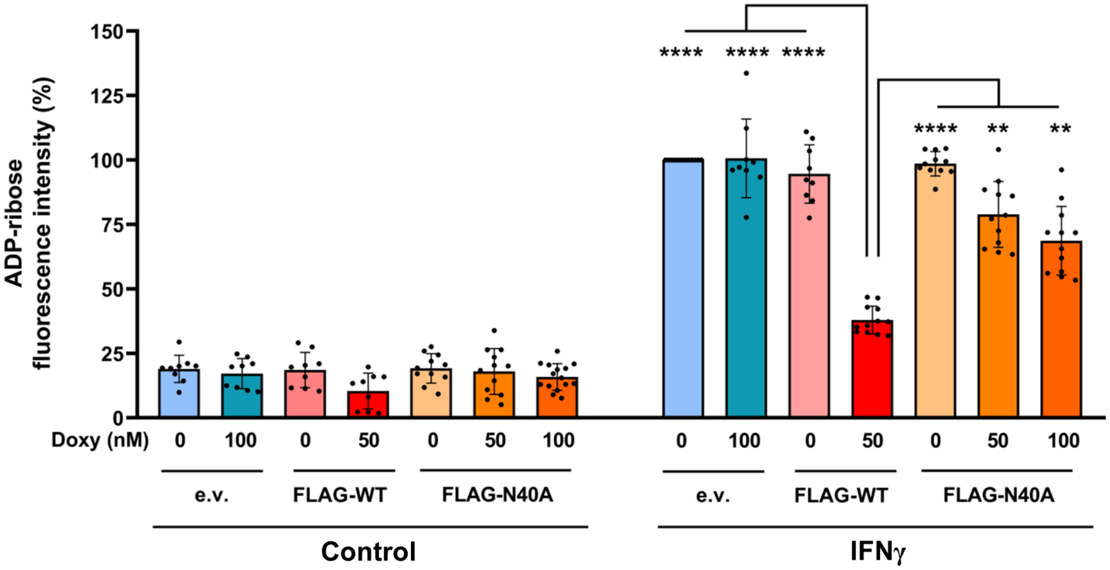

Supplementary Figure 2 (related to Figure 2)

(A) Representative image of immunoblot analyses for FLAG and actin loading control in A549 cells transduced with empty vector control (e.v.) or with lentiviral constructs for constitutive expression of FLAG-tagged WT or N40A mutant macrodomain, 24h after treatment with vehicle control, 1000 U/mL IFNα, 1000 U/mL IFNβ, 100 U/mL IFNγ or transfected with 0.1 μg/mL poly(I:C). Same experiment as Main Figure 5C, showing same FLAG panel with additional lanes. Actin loading control from the same membrane as FLAG.

(B) Example of the gating procedure used to normalize FLAG macrodomain expression levels. (left) Mean FLAG immunofluorescence intensity in A549 cells transduced with empty vector control (e.v.) or with lentiviral constructs for constitutive expression of FLAG-tagged WT or N40A mutant macrodomain. (middle) Histograms of the FLAG signal intensity of the cell populations and gating strategy (red box) to select cells with comparable levels of macrodomain expression. (right) Mean FLAG immunofluorescence intensity in gated cells, showing comparable levels of expression of cells within this population, used for analyses in Main Figure 2C.

(C) Re-analysis of the results shown in Main Figure 2C using all cells, without the gating strategy described in Sup.Fig.2B.

(D) Representative images of the data shown in Main Figure 2C and Sup. Fig. 2C. Scale bars = 20 μm

(E) Representative image of immunoblot analyses for FLAG and actin loading control in A549 cells transduced with empty vector control (e.v.) or lenvitival constructs for doxycycline-inducible expression of FLAG-tagged WT or N40A mutant macrodomain, 24h after treatment with 100 U/mL IFNγ and indicated doses of doxycycline.

(F) Example of the gating procedure to normalize FLAG macrodomain expression between cell populations, similar to Sup.Fig. 2B above.

(G) Re-analysis of the results shown in Main Figure 2D using all cells, without the gating strategy shown in Sup.Fig. 2F.

Supplementary Figure 3

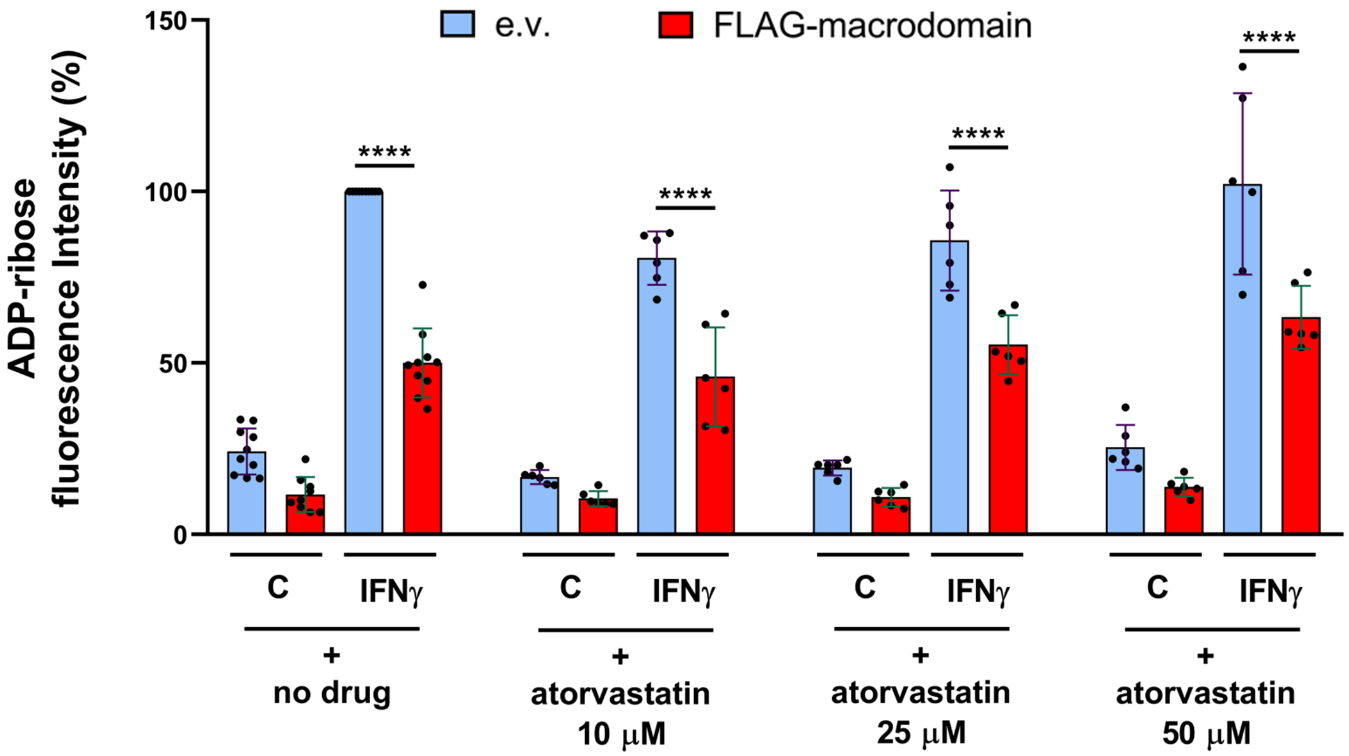

Supplementary Figure 3 (related to Figure 3)

(A) Quantification of ADP-ribose immunofluorescence signal intensity in A549 cells transduced either with empty vector control (e.v.) or with a lentiviral construct for constitutive expression of WT macrodomain, 24h after treatment with vehicle control, 100 U/mL IFNγ or 100 U/mL IFNγ + indicated doses of atorvastatin. Mean ± SEM (n=6, from 3 separate experiments). ****=p<0.0001.

Supplementary Figure 4

A

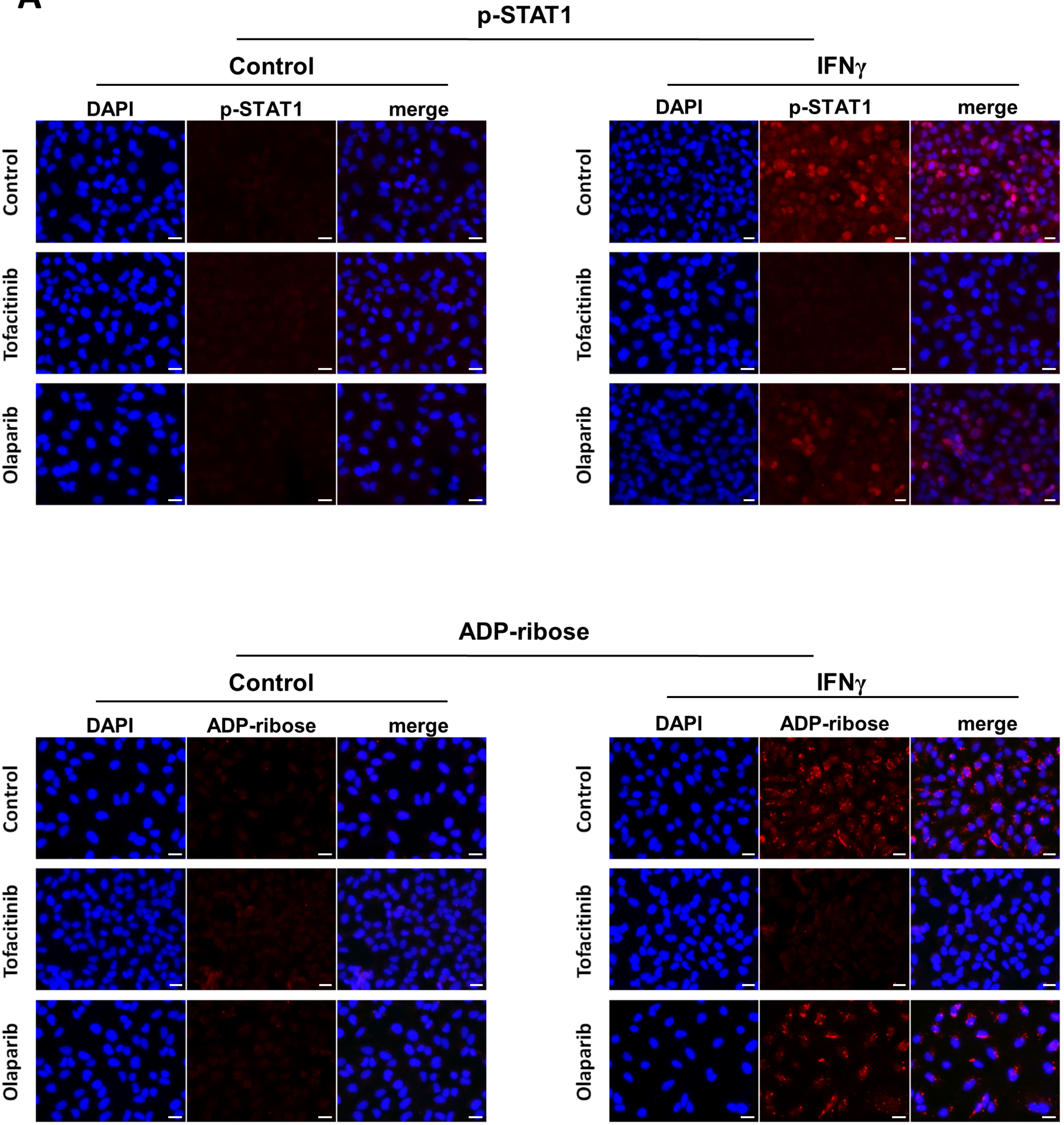

Supplementary Figure 4

B

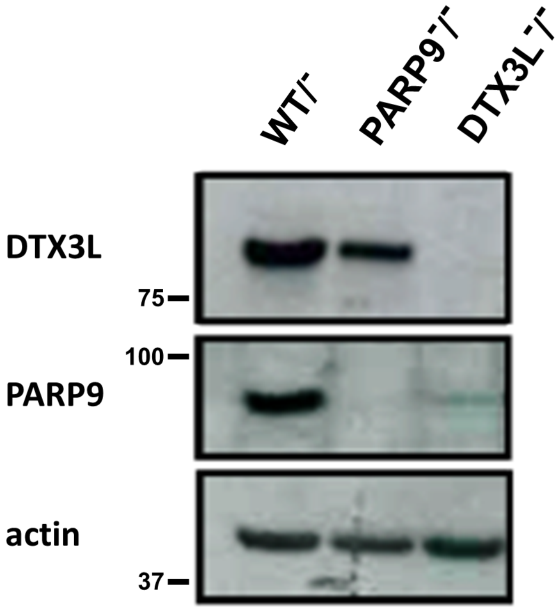

C

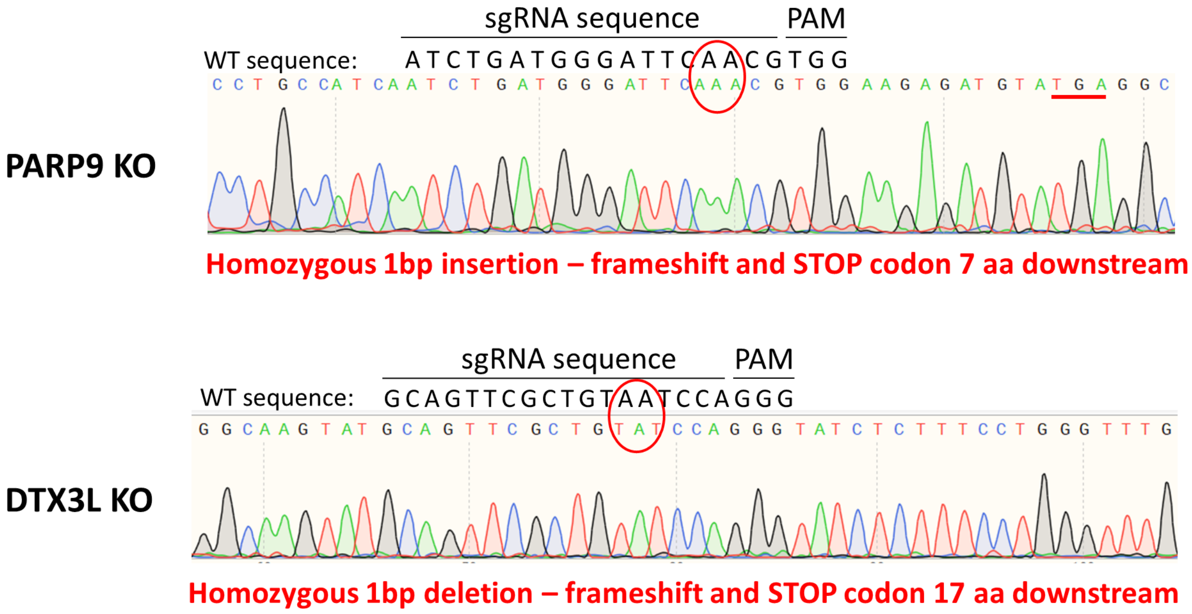

Supplementary Figure 4

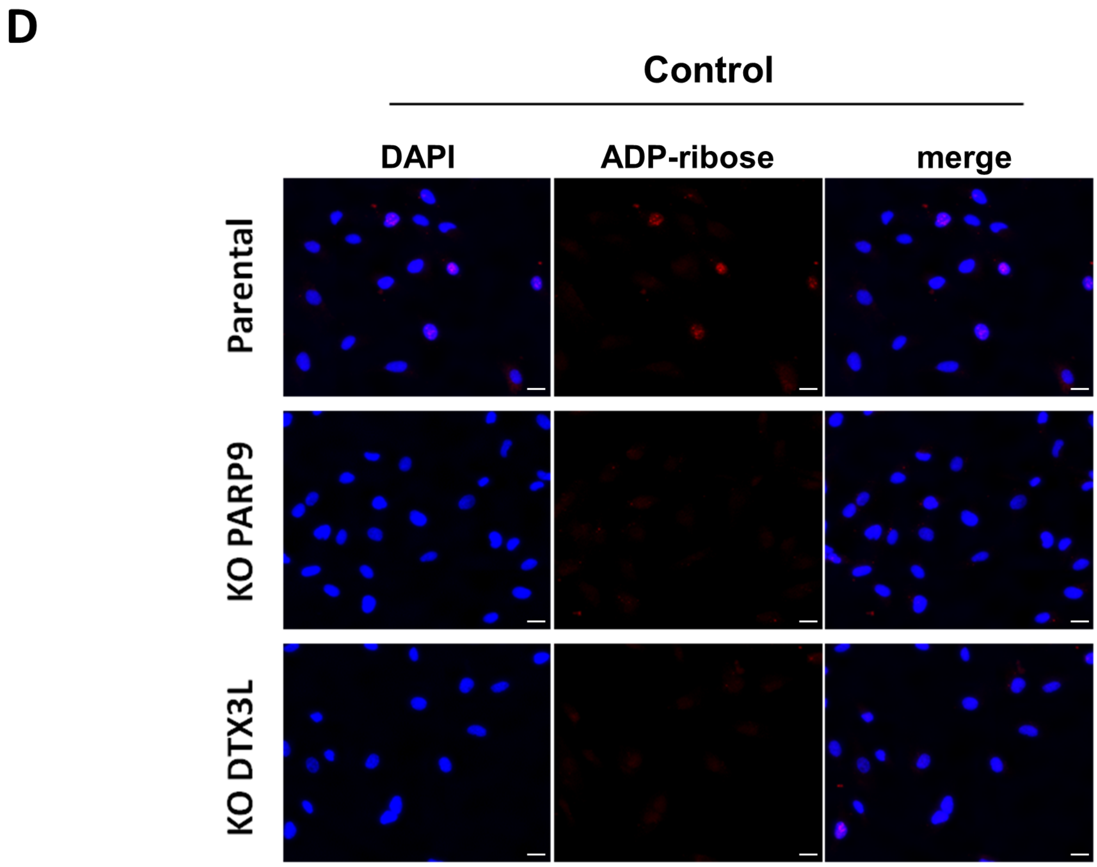

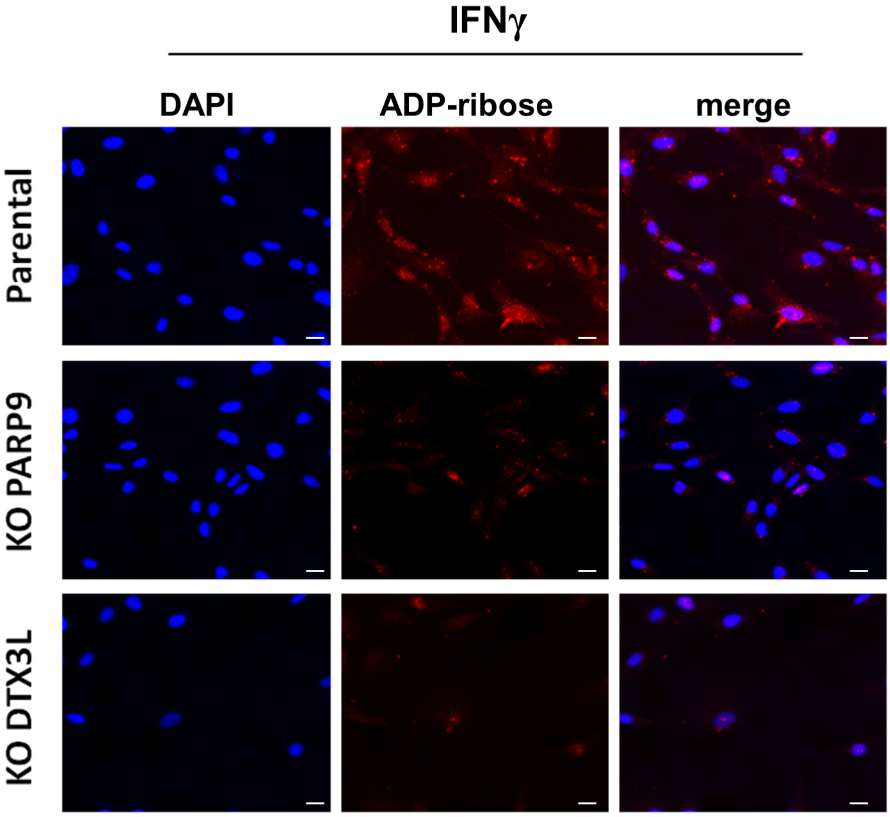

Supplementary Figure 4 (related to Figure 4)

(A) Representative images of the results presented in Main Figures 4A and 4B. Scale bars = 20 µm.

(B) Representative image of immunoblot analyses for PARP9, DTX3L and actin loading control in RPE1-hTERT WT, PARP9 KO and DTX3L KO cells used in this study.

(C) Sanger sequencing traces obtained from PCR products of the genomic DNA surrounding the gRNA target locus in the PARP9 KO clone (top) and DTX3L KO clone (bottom) used in this study. The WT sequence and PAM location are shown above each trace for comparison, and the indel is highlighted with a red circle. The premature stop codon in the shifted reading frame caused by the indel detected in the PARP9 KO cells is underlined. Both clones are apparently homozygous for the shown allele, as the PCR product is expected to contain a mixture of amplicons from both alleles.

(D) Representative images of the results presented in Main Figure 4C. Scale bars = 20 µm.

Supplementary Figure 5

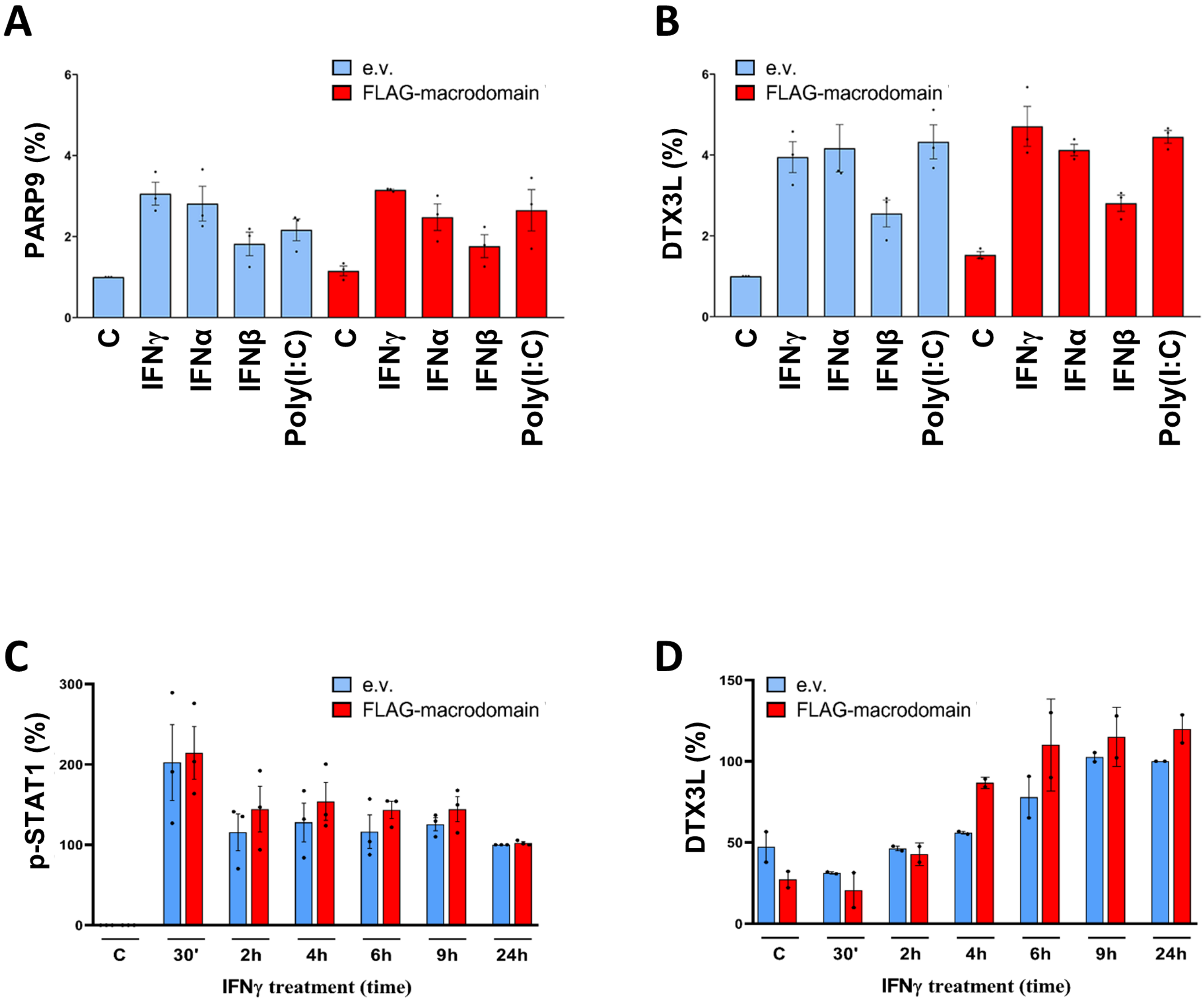

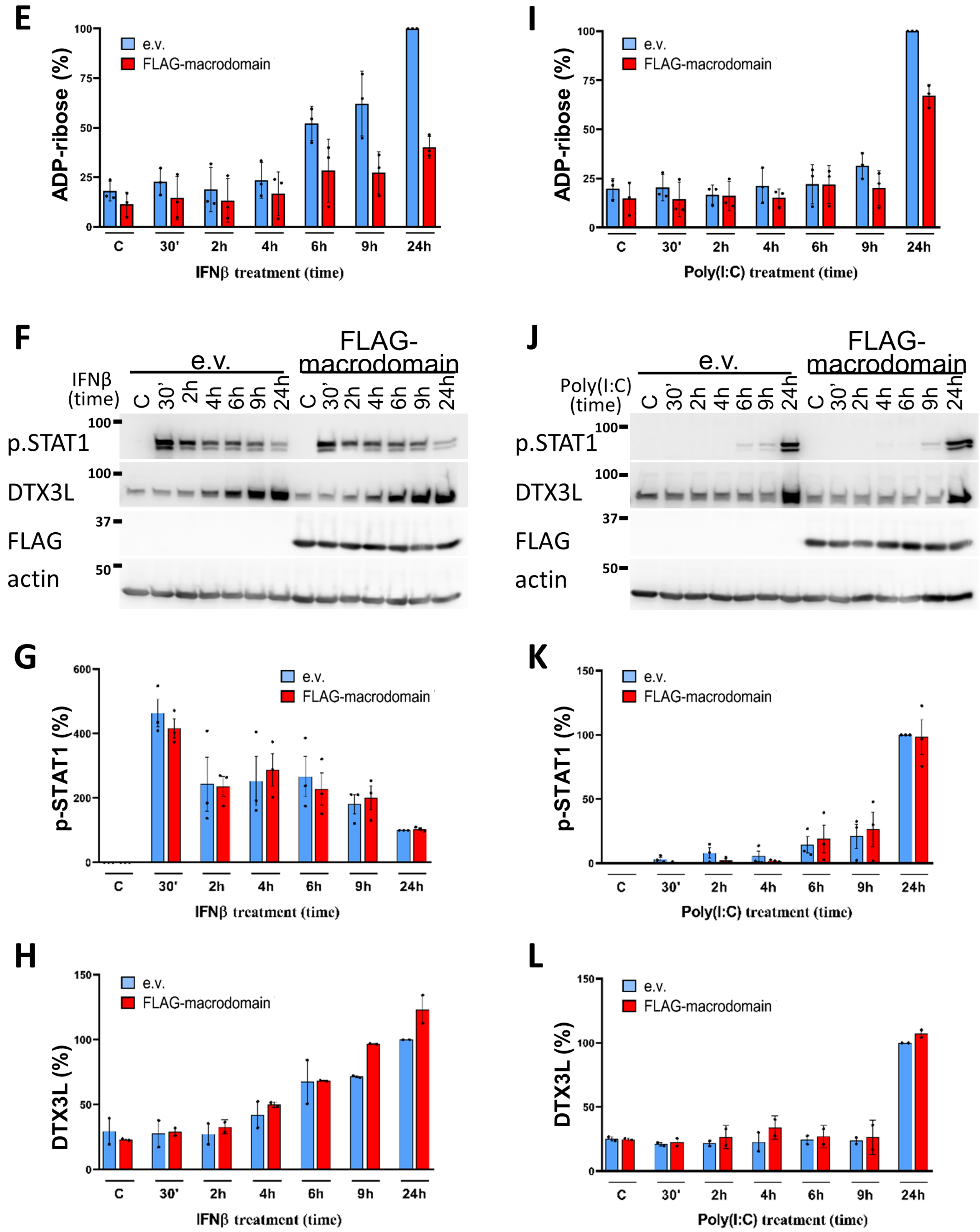
E
ADP-ribose (%)
e.v.
FLAG-macrodomain
C
30'
2h
4h
6h
9h
24h
IFNβ treatment (time)
I
ADP-ribose (%)
e.v.
FLAG-macrodomain
Poly(I:C) treatment (time)
F
IFNβ (time)
e.v.
FLAG-macrodomain
p.STAT1
DTX3L
FLAG
actin
J
Poly(I:C) (time)
e.v.
FLAG-macrodomain
p.STAT1
DTX3L
FLAG
actin
G
p-STAT1 (%)
e.v.
FLAG-macrodomain
IFNβ treatment (time)
K
p-STAT1 (%)
e.v.
FLAG-macrodomain
Poly(I:C) treatment (time)
H
DTX3L (%)
e.v.
FLAG-macrodomain
IFNβ treatment (time)
L
DTX3L (%)
e.v.
FLAG-macrodomain
Poly(I:C) treatment (time)

Supplementary Figure 5 (related to Figure 5)

(A) Quantification of PARP9 protein levels and **(B)** quantification of DTX3L protein levels from the immunoblot analyses shown in Main Figure 5C. Mean ± SEM (n=3).

(C) Quantification of STAT1 phospho-Y701 and **(D)** quantification of DTX3L protein levels from the immunoblot analyses shown in Main Figure 5E. Mean ± SEM (n=3).

(E) Quantification of ADP-ribose immunofluorescence signal intensity, **(F)** representative image of immunoblot analyses for STAT1 phospho-Y701, DTX3L, FLAG and actin loading control, **(G)** quantification of STAT1 phospho-Y701 immunoblots and **(H)** quantification of DTX3L immunoblots in A549 cells transduced either with empty vector control (e.v.) or lentiviral constructs for constitutive expression of FLAG-tagged macrodomain, after treatment with 1000 U/mL IFNβ for the indicated times. Mean ± SEM (n=3).

(I-L) Same as **(E-H),** except that cells were stimulated with 0.1 μg/mL poly(I:C). Mean ± SEM (n=3).

Supplementary Tab1e 1 - Compounds tested in the repurposing screen

compound number	compound name	ZINC15 code	Concentration (μM) used in Fig. 3C*
#1	fexofenadine	ZINC000003872566	50
#2	cefamandole	ZINC000003830394	50
#3	Pralatrexate	ZINC000001536109	50
#4	aminopterin	ZINC000002036915	50
#5	ticagrelor	ZINC28957444	10
#6	Leucovorin	ZINC000009212428	50
#7	pravastatin	ZINC000003798763	50

#8	cefonicid	ZINC000003830428	50
#9	latanoprostene bunod	ZINC95598449	50
#10	atorvastatin	ZINC000003932191	10
#11	tartrazine	ZINC100048501	50
#12	domperidone	ZINC4175569	10
#13	Telmisartan	ZINC1530886	50
#14	eltrombopag	ZINC11679756	10
#15	Netarsudil	ZINC113149554	1
#16	Terfenadine	ZINC000003831511	1
#17	vilazodone	ZINC000001542113	10
#18	perospirone	ZINC13828184	50
#19	bazedoxifene	ZINC1895505	10
#20	axitinib	ZINC3816287	50
#21	panobinostat	ZINC000022010649	0.1
#22	abemaciclib	ZINC72318121	1
#23	hydroxyhaloperidol	ZINC4257980	50
#24	telotristat	ZINC84758235	10
#25	Saquinavir	ZINC26985532	10
#26	cabozantinib	ZINC70466416	10
#27	bromperidol	ZINC601270	10
#28	tiagabine	ZINC000003831531	50
#29	chlorhexidine	ZINC000014768621	1
#30	diclofenac acyl glucuronide	ZINC000035048352	50
#31	rosiglitazone, n-desmethyl	ZINC000022056375	50
#32	cisapride	ZINC3830564	50

#33	enalaprilat	ZINC000003812851	50
#34	troglitazone	ZINC968278	50
#35	Daidzin	ZINC000004098610	50
#36	Tauroursodeoxycholic acid	ZINC000003914813	50
#37	7-hydroxymethotrexate	ZINC000008655696	50
#38	Terconazole	ZINC3873936	1
#39	5-o-desmethyldonepezil	ZINC13449462	50
#40	apixaban	ZINC11677837	50
#41	avobenzone	ZINC973	50
#42	dolutegravir	ZINC58581064	50
#43	luteolin	ZINC000018185774	10
#44	tranilast	ZINC000000000797	50
#45	Ataluren	ZINC000013831791	50
#46	omeprazole	ZINC000005116154	50
#47	dantrolene	ZINC000002568036	50
#48	rutaecarpine	ZINC000000898237	10
#49	flubendazole	ZINC000003830847	50
#50	Tiaprofenic acid	ZINC2282	50
#51	nitazoxanide	ZINC000003956788	50
#52	cephapirin	ZINC000003830511	50
#53	tofacitinib	ZINC3818808	50
#54	(+/-)-Sulfinpyrazone	ZINC2004425	10
#55	Carmofur	ZINC1542916	50
#56	Chloramphenicol	ZINC113382	50
#57	Droperidol	ZINC19796080	50

#58	Enzalutamide	ZINC34806477	50
#59	Fludarabine Phosphate	ZINC3927870	50
#60	Idelalisib	ZINC13986658	50
#61	Isoliquiritigenin	ZINC3869608	10
#62	Meropenem	ZINC3808779	50
#63	Pentostatin	ZINC3806262	50
#64	Pirbuterol	ZINC637	50
#65	Safinamide	ZINC53084692	50
#66	Tizoxanide	ZINC5924265	50
#67	Tobramycin	ZINC8214692	50
#68	Vidarabine	ZINC970363	50
#69	Vorinostat	ZINC1543873	1

*Highest concentration for each compound that allowed >90% survival, as measured by a preliminary MTT assay.
